# Nanomaterials for smart wearable fibers and textiles: A critical review

**DOI:** 10.1016/j.isci.2025.113126

**Published:** 2025-07-16

**Authors:** Linlin Zhu, Weiyi Zhang, Shuo Luan, Jiaqi Wei, Yang Yang, Jinlei Miao

**Affiliations:** 1 School of Materials and Chemical Engineering, Bengbu University, Bengbu, Anhui 233030, China; 2 College of Textiles & Clothing, Qingdao University, Qingdao 266071, P.R. China; 3 Anhui Fengyuan Biotechnology Co, Ltd, Bengbu 233700, China; 4 Textile Ecological Dyeing and Finishing Key Laboratory of Sichuan Province, Chengdu Textile College, Chengdu, Sichuan 610097, P.R. China

**Keywords:** Health sciences, Chemistry, hysics, Materials science

## Abstract

Smart wearable devices with functionality and comfort are highly anticipated in practical applications for health monitoring, personal thermal management, and electromagnetic shielding. As an important branch of wearable devices, smart wearable fibers and textiles with high breathability and comfortability are eagerly desired for practical wearable applications, which have revolutionized the development of smart apparel. The introduction of nanomaterials (including carbon nanotubes, graphene, MXene, conductive polymers, and metal nanowires) with unique mechanical, electrical, and chemical properties can not only improve the functionality of wearables, but also maintain the flexibility of wearables. This review comprehensively summarizes recent advances in nanomaterial-based fibers and textiles for smart wearables, including the state-of-art fabrication methods, properties, and wearable applications. The role of nanomaterials in enhancing mechanical, electrical, and smart wearable versatility is highlighted, as well as the challenges and future directions for smart wearable fibers and textiles.

## Graphical abstract

**Figure:**
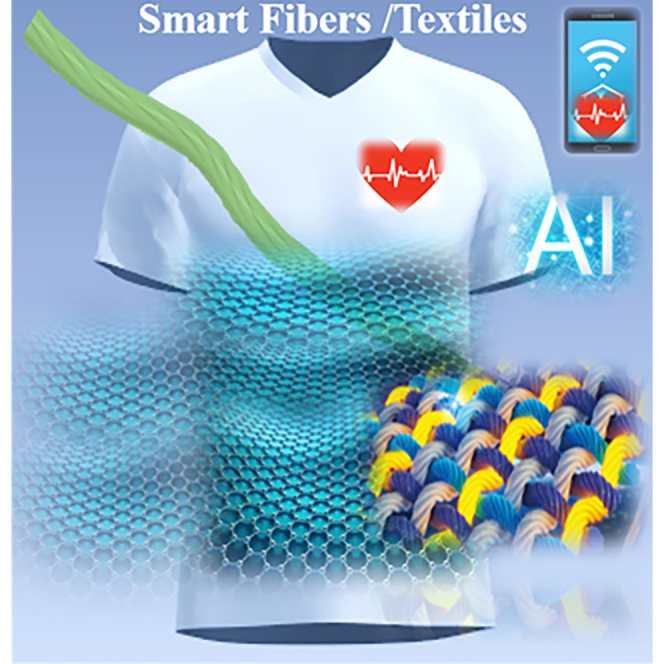

## Teaser

Health sciences; Chemistry; Physics; Materials science

## Introduction

Flexible electronic products, such as bendable/foldable touch screens, curved light-emitting diodes/solar cells, soft electronic skins, stretchable energy conversion and storage devices, have attracted much great attention in recent years.^1^^,^^2^^,^^3^ Compared with traditional rigid and bulky electronic devices, lightweight, flexible, and wearable electronic devices with high mechanical deformation ability and adaptability can seamlessly adapt to the curved skin and intense activities of the human body, and provide a comfortable wearing experience.^4^ Especially, along with the rapid development of AI and the Internet of Things (IoT), there is a growing demand for flexible electronic products, as they can naturally adhere to human skin and continuously collect real-time human data such as blood sugar, pressure, heart rate, and pulse.^5^ Meanwhile, emerging wearable technology as a disruptive technology that explores new ways of interaction between humans and technology. It can cross integrate with key core technologies such as communication technology, computer technology, AI, materials science, life and health, and intelligent sensing, leading technological innovation and transformation, and driving related industries to achieve new leapfrog development. Currently, wearable technology, as a national strategic emerging technology, has entered a period of explosive growth, presenting unprecedented application potential in industries such as industry, military, healthcare, education, and entertainment.

In order to promote the booming development of flexible electronics and wearable technology, efficient healthcare, and thermal comfort management during long-term wear are fundamental requirements for maintaining health and avoiding thermal runaway in the human body, which is also a basic requirement for all wearable devices.^6^^,^^7^ People urgently hope to develop wearable electronic products with self-perception and self-regulation capabilities, as the “second layer of skin” of the human body, which can effectively monitor human health status and regulate thermal comfort.^8^ Conventional wearable electronics, such as electronic skins (E-skins) or film-based flexible electronics, are mainly based on elastic films such as rubber and elastomers, which are impermeable and prone to skin inflammation and diseases.^9^ Breathability is an inevitable requirement for wearable electronics and systems. Although current fibers/fabrics made of natural and synthetic polymers have higher breathability and much more comfort compared to e-skin and film-based wearable electronics, they are essentially electrically/thermally insulated, which defies the sensing and conditioning principles of wearable electronics that rely on electronic hardware/software for signal transmission, making it difficult to develop them into wearable electronic devices. Fortunately, the rapid development of nanomaterials in recent years and their integration into wearable fibers/textiles to build conductive networks can endow the wearables with functions such as conductivity and sensing, revolutionizing the development of smart multifunctional systems capable of sensing, energy harvesting, and health monitoring.^10^^,^^11^^,^^12^ Nanomaterials, with their unique size-dependent properties, have emerged as critical components in enabling these functionalities. Nanomaterials such as carbon nanotubes (CNTs), graphene, MXene, and silver nanowires are pivotal for enhancing the mechanical, electrical, and chemical properties of fibers and textiles.

Introducing flexible low dimensional conductive materials into fibers to construct flexible conductive networks, in order to explore lighter, stronger, tougher, and more conductive/thermally conductive fibers, is considered an ideal strategy to develop smart wearable fibers and textiles.^12^^,^^13^^,^^14^^,^^15^ In another way, despite exciting progress and the development of various flexible conductive candidate materials in recent years, including conductive polymers, metal nanoparticles, metal nanowires, carbon-based materials such as carbon nanotubes and graphene, to construct conductive networks in fibers, their performance still lags behind the demand for practical wearable applications.^16^ For example, the severe aggregation of metal particles and carbon-based materials, the low electrical conductivity of conductive polymers, the poor environmental stability of metal nanowires, the poor dispersion and high contact resistance of carbon-based materials result in significantly lower performance than expected.^17^^,^^18^^,^^19^^,^^20^^,^^21^ In addition, when low dimensional materials such as nanoparticles, nanotubes, nanowires, and nanosheets are assembled from microscopic building blocks into macroscopic networks, the numerous overlapping interfaces and weak interface contacts between them not only lead to the failure of the response network under stress (such as fracture, damage, and detachment from the fiber surface),^22^ but also seriously reduce the stretchability, sensitivity, and monitoring signal reliability of their wearable electronic products.^23^

**Figure 1:**
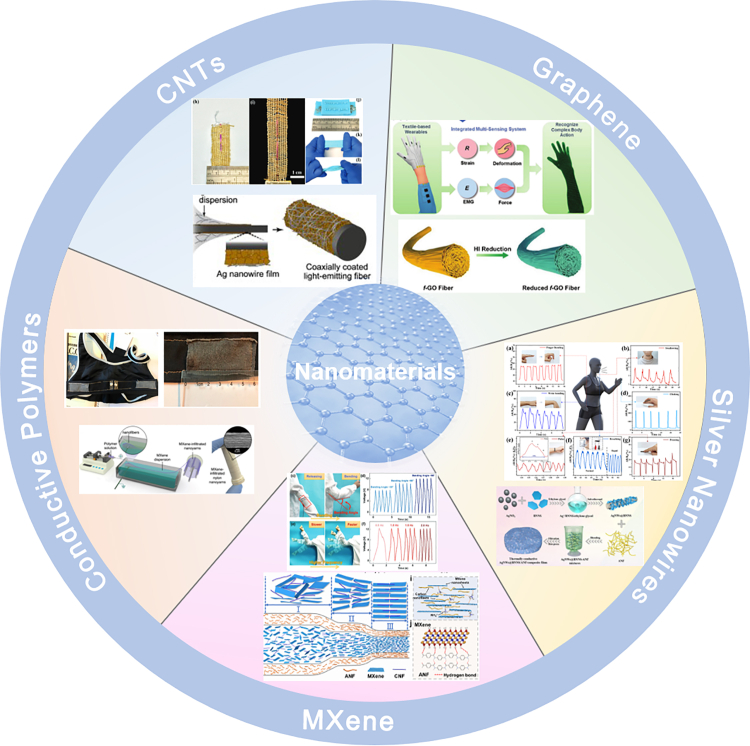
Nanomaterials for smart wearable fibers and textiles

In this review, we would summarize the properties and recent research progress of various conductive nanomaterials for smart wearable fibers and textiles, all of which own excellent electrical conductivity, thermal stability and other advantages, as well as hold great promise in smart wearable devices. Furthermore, depending on different preparation strategies, smart wearable fibers and textiles will have excellent performance to meet various wearable applications demands. Hence, this review provides a unique perspective of the research progress of smart wearable fibers and textiles based on nanomaterials in recent years, analyzing and summarizing the fabrication principle of the wearable systems and the important performance parameters (Figure 1). Furthermore, the core elements of smart wearable fibers and textiles are described in details, mainly including the research and application of high-performance conductive materials and wearable system. Meanwhile, the review lists some common fabrication strategies and research progress of smart wearable fibers and textiles. Finally, the development trend of the smart wearable fibers and textiles is summarized and prospected, and the critical opportunities and challenges are also further discussed.

## Conductive nanomaterials

### Graphene

Graphene is a 2D carbon nanomaterial composed of *sp*^2^ hybridized orbitals forming a hexagonal honeycomb lattice. So far, graphene is the thinnest and strongest nanomaterial, with a thin sheet thickness of 0.34 nm. The structure of graphene is very stable, with a C-C bond length of only 0.142 nm.^24^ The connections between each carbon atom in graphene are very strong, and when external force is applied to graphene, the atomic surfaces inside the graphene deform and further bend to counteract the external force. Therefore, there is no rearrangement or displacement between carbon atoms, maintaining a consistently stable structure. Nowadays, there are many methods for preparing graphene, but the main methods include mechanical exfoliation, liquid-phase exfoliation, chemical vapor deposition, epitaxial growth, and redox methods, which have excellent optical, electrical, and mechanical properties.^19^^,^^25^^,^^26^ Graphene has a Young’s modulus of 1.0 Tpa and a tensile strength of 130 Gpa, making it one of the strongest materials known. It also has good toughness and can bend, with an elastic modulus of up to 1 Tpa. Graphene has low electrical resistivity (∼10^−6^ Ω·cm) and high carrier mobility (15,000 cm^2^·V^−1^·s^−1^). Unlike traditional conductive materials, the electron mobility of graphene is less affected by changes in ambient temperature. Graphene has excellent thermal conductivity, with pure graphene having a thermal conductivity coefficient of up to 5300 W·mK^−1^, which is the highest among existing carbon materials, far higher than single-walled carbon nanotubes (3,500 W·mK^−1^) and multiwalled carbon nanotubes (3,000 W·mK^−1^). Compared to pristine graphene, which exhibits superior electrical conductivity (10^6^ S·m^−1^), reduced graphene oxide (rGO) and graphene oxide (GO) demonstrate significantly lower conductivities (∼10^3^ S·m^−1^ and insulating properties, respectively). The hydrophilicity of GO facilitates its uniform coating on textiles, whereas the intrinsic hydrophobicity of pristine graphene necessitates surfactant-assisted dispersion, often leading to aggregation issues. Pristine graphene/rGO coatings suffer from weak adhesion to textile substrates, resulting in detachment after repeated washing cycles. In contrast, CVD-grown graphene offers enhanced stability but compromises breathability due to its dense, impermeable structure.

**Figure 2:**
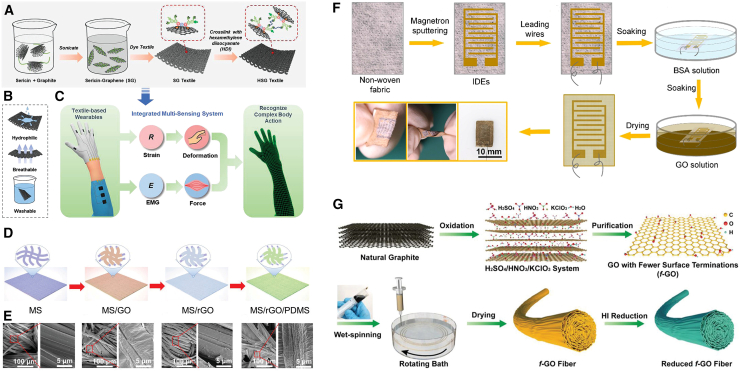
Application of graphene in smart wearable fibers and textiles (A–C) Fabrication schematic (A) and main characteristics of hexamethylene diisocyanate cross-linked sericin-graphene (B; HSG), and an integrated multi-sensing system based on HSG textiles (C).^21^ Copyright (2022), with permission from John Wiley and Sons. (D) The manufacturing process of waterproof and breathable graphene-based electronic fabrics. (E) SEM images of surface topography of MS, MS/GO, MS/rGO, and MS/rGO/PDMS.^22^ Copyright (2022), with permission from John Wiley and Sons. (F) Schematic diagram of the device manufacturing process.^28^ Copyright (2020), with permission from American Chemical Society. (G) Schematic diagram of the preparation process of f-GO, f-GO fibers, and reduced f-GO fibers.^29^ Copyright (2022), with permission from John Wiley and Sons.

Graphene has excellent flexibility, good conductivity, and stability, making it an ideal material for constructing electronic fabrics.^27^ However, it is difficult to prepare water-based conductive inks based on hydrophobic graphene. Liang et al.^21^ prepared water-based graphene conductive ink with the assistance of natural sericin, overcoming the aforementioned drawbacks. Furthermore, the obtained electronic fabric based on graphene ink is hydrophilic, breathable, and washable, and can monitor and recognize complex human activities, transforming them into signal reflections, providing a promising research prospect for manufacturing intelligent fabric systems (Figure 2A–2C). Wang et al.^22^ prepared a waterproof and breathable knitted electronic skin by loading GO and further reducing it into graphene, and finally encapsulating it with polydimethylsiloxane (Figures 2D and 2E). The obtained electronic skin has excellent self-cleaning performance and is not contaminated by most stains in daily life, such as tea, coffee, etc. Furthermore, the electronic fabric can also monitor human movement, providing new research ideas for intelligent wearable electronic fabrics.

Due to the chemical inertness of graphene, it is difficult to effectively disperse it in solvents for large-scale solution treatment. GO has attracted widespread attention due to its advantages such as low production cost, large-scale production, and easy processing. Oxidized graphene is the product of graphene oxidation, which still maintains the layered structure of graphite, but with an increase in oxygen-containing functional groups, its properties become more active. GO has superior hydrophilicity and can be well dispersed in water. GO was prepared by hummers method, with an average modulus of up to 0.25 TPa and a large specific surface area. Through high-temperature reduction or chemical reduction, structurally stable reduced GO (rGO) conductive materials can be obtained.^23^ As shown in Figure 2F, Wang et al.^28^ developed a highly sensitive humidity sensor by coating GO on non-woven fabric (NWF) and improved the adsorption of GO on NWF using bovine serum albumin. This humidity sensor not only has high humidity sensitivity, but also can monitor respiratory behavior under different conditions and make rapid responses, which is a promising research for human healthcare. By optimizing the surface chemistry of oxidized graphene and controlling spinning and assembly, as shown in Figure 2G, Tang et al.^29^ studied and prepared a scalable wet spun graphene fiber with strong toughness and excellent conductivity. The tensile strength of graphene fibers obtained by wet spinning is 791.7 MPa, and the toughness and conductivity reach 8.3 MJ·m^−3^ and 1.0 × 10^5^ S·m^−1^, respectively. This study provides a new approach for the large-scale production of high-performance graphene fibers and flexible wearable devices.

Although graphene has emerged as a pivotal material for smart textiles due to its exceptional electrical conductivity (10^6^ S·m^−1^), mechanical strength (tensile strength of 130 GPa), and flexibility, its practical implementation faces multiple challenges. Such as chemical vapor deposition (CVD) yield high-quality pristine graphene but are costly and incompatible with large-scale textile production. In contrast, liquid-phase exfoliation and Hummers-derived GO are scalable and cost-effective but compromise conductivity due to structural defects and residual oxygen groups. RGO partially restores conductivity but requires harsh chemical treatments, raising environmental concerns.

### Carbon nanotubes

Carbon nanotubes (CNTs), as a special 1D nanotube material, which can be considered as coiled from layered graphene nanosheets, lightweight, and perfectly connected in a hexagonal structure. Carbon nanotubes have excellent mechanical, electrical, and chemical properties, and have broad application prospects. CNTs is widely used in wearable devices due to its high aspect ratio, extremely low density, excellent flexibility, mechanical properties, and chemical stability.^30^^,^^31^

**Figure 3:**
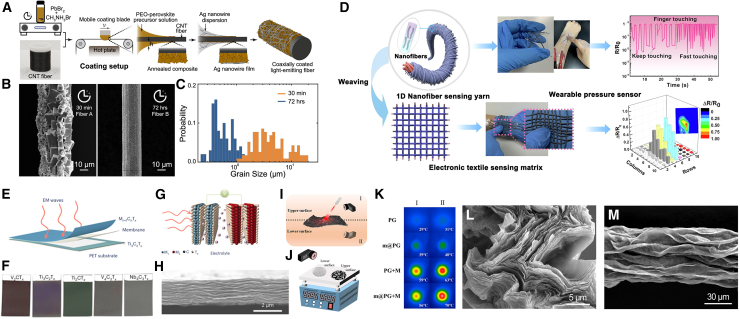
Application of carbon nanotubes and MXene in smart wearable fibers and textiles (A) Schematic diagram of the fabrication process for coaxially coated luminescent carbon nanotube fibers. (B) SEM images of the perovskite-PEO composite film. (C) Size distribution of microcrystals formed on Fiber A and Fiber B, extracted from the image shown in (B).^32^ Copyright (2020), with permission from American Chemical Society. (D) Schematic diagram of the fabrication process for the core-sheath nanofiber pressure sensor.^33^ Copyright (2020), with permission from Elsevier. (E) Schematic of the shielding structure, composed of MXene electrodes on a PET substrate and a polymer membrane containing electrolyte. (F) Photograph of the MXene electrodes used. (G) Schematic diagram of ion intercalation between MXene layers for tuning EMI shielding performance. (H) Cross-sectional SEM image of the MXene film.^35^ Copyright (2023), with permission from Springer Nature. (I) Schematic of the infrared imaging setup detecting the fiber membrane at two positions: I and II. (J) Schematic of the infrared imaging monitoring for the upper and lower surfaces of the fiber membrane placed on a hot plate. (K) Temperature distribution of the fiber membrane after 1-min irradiation at positions I and II.^36^ Copyright (2023), with permission from Elsevier. (L) Typical SEM image of the Ac-MXene fiber. (M) Typical SEM image of the Ac-MXene fiber in cross-sectional view.^37^ Copyright (2023), with permission from American Chemical Society.

The Jamali team applied a precursor solution of polyethylene oxide (PEO) – methyl lead bromide (MAPbBr_3_) onto carbon nanotube-based fibers through a layer-by-layer solution coating method, successfully preparing CNTs-based luminescent fibers.^32^ The surface layer of the luminescent fiber is formed by coating silver nanowire dispersion after cooling and annealing (Figures 3A–3C), and the core is composed of highly elastic flexible fibers and CNTs fibers. Organic inorganic composite layers are alternately added between the core and the surface layer, adding many functions to the luminescent fiber. Qi et al.^33^ utilized the high elasticity and conductivity of nickel-plated cotton fibers as the core layer, and prepared CNTs-based nanofiber yarns through electrospinning (Figure 3D). This yarn has a core-shell structure, with a shell layer of polyurethane (PU) nanofibers embedded with CNTs. Therefore, the sensing sensitivity of this yarn is extremely high, and it has extremely high comfort. It can closely adhere to human skin without any foreign object sensation, making it an ideal material for flexible electronic fabrics. Hu team^34^ prepared a multifunctional carbon nanotube-based aerogel film by incorporating CNTs into ANF and hydrophobically modifying it with fluorocarbon resin. The prepared multifunctional aerogel film has excellent hydrophobicity (WCA = 137.0°), large specific surface area (232.8 m^2^·g^−1^) and good flexibility, while exhibiting a high conductivity (230 S·m^−1^) to realize the good Joule thermal effect and electromagnetic shielding performance, which has received extensive research by researchers of smart wearable, personal thermal management and electromagnetic shielding. The study has received wide attention from researchers in smart wear, personal thermal management and electromagnetic shielding.

Despite their potential in wearable devices, carbon nanotubes still face a number of problems; CNTs synthesized using CVD methods are costly, and CNTs prepared using low purity arc discharge derivatization contain metallic impurities that impair sensor linearity. Meanwhile, smart wearable devices prepared by coating methods with bare CNTs coatings that are susceptible to degradation under UV irradiation and humid environments require protective polymer encapsulation, which tends to impair flexibility and increase cost. CNTs fibers prepared by wet spinning, on the other hand, require complex post-treatment processes. The selection of appropriate preparation methods is of great importance to improve the application of carbon nanotubes in wearable devices.

### MXene

MXene is a 2D layered inorganic compound composed of transition metal nitrides and carbides (MXenes). Due to its excellent conductivity, thermal conductivity, mechanical and chemical properties, as well as wide potential applications, it has been widely developed as an emerging family of 2D materials.^38^^,^^39^^,^^40^ MXenes have a general configuration of M_n+1_X_n_T_x_, where M, X, and T represent transition metals, carbon/nitrogen, and surface end functional groups such as O, F, and OH, respectively. By selectively etching the MAX phase with acid, the M-A bond, which is more chemically unstable than the M-X bond, is broken, resulting in multilayer MXene (*m*-MXene) with an accordion-like structure. Due to the loose and easy to peel off structure of multilayered MXene, 2D MXene nanosheets with a single layered structure and stable dispersion can be obtained through methods such as ultrasound.^41^^,^^42^^,^^43^^,^^44^

MXene nanosheets have excellent electromagnetic shielding effects. To protect electronic devices operating at higher frequencies, Han et al.^35^ developed a method that utilizes multiple submicron sized MXene nanosheets to control the reflection and absorption of incident electromagnetic waves, thereby achieving reversible and adjustable electromagnetic interference shielding effects (Figures 3E–3H). Compared with static electromagnetic interference (EMI) shielding, the developed MXene film achieves active regulation through electrochemical driven ion intercalation and delamination, which can adapt to more harsh environments. Ding et al.^36^ prepared MXene composite nanofibers with thermal management function using electrospinning technology (Figures 3I–3K). By utilizing the generated unidirectional thermal conductivity, they can be used as wound dressings after skin cancer resection surgery, eliminating residual cancer cells and inhibiting their growth through photothermal therapy. This study provides new ideas for directional heat transfer and has broad application prospects in postoperative care. Zuo et al.^45^ used aramid aerogel fiber as a porous framework, loaded 1D silver nanowires and 2D MXene nanosheets, and successfully prepared smart fibers with a variety of external stimulus responses (mechanical, electrical, thermal and optical signals), which provided an efficient strategy for outdoor travel and continuous operation of the lunar rover, and also brought great hope for the next generation of self-powered wearable systems. Zheng et al.^37^ developed a novel interface crosslinking method to prepare MXene fibers with high flexibility and electrochemical properties (Figures 3L and 3M). The obtained MXene fibers have high conductivity (3545 S·cm^−1^), mechanical strength (205.5 MPa), and high pseudocapacitive charge storage capacity (1570.5 F·cm^−3^). The assembled fiber capacitor can still maintain 99.5% capacitance under mechanical deformation, and can be further integrated into textiles to power electronic devices. This work provides a new approach for the manufacturing of advanced MXene fibers and the development of high-performance flexible fiber supercapacitors. In summary, the significant inherent properties of MXene make it widely used in various fields such as wearable sensors, energy storage devices (lithium-ion batteries, sodium ion batteries, and supercapacitors), and electromagnetic shielding.

Although MXene has tunable surface chemistry and high specific capacitance, it has become an ideal material for smart textiles. However, the etching process of MXene is hazardous and the yield is low, which makes it difficult to produce on a large scale. In addition, MXene is easily oxidized in air, which can reduce its conductivity by more than 50%, so its long-term stability in the environment remains to be verified. Therefore, it is necessary to solve the problem of MXene to find a suitable preparation method to improve the environmental durability and stability of its performance in the field of smart textiles.

### Conductive polymers

**Figure 4:**
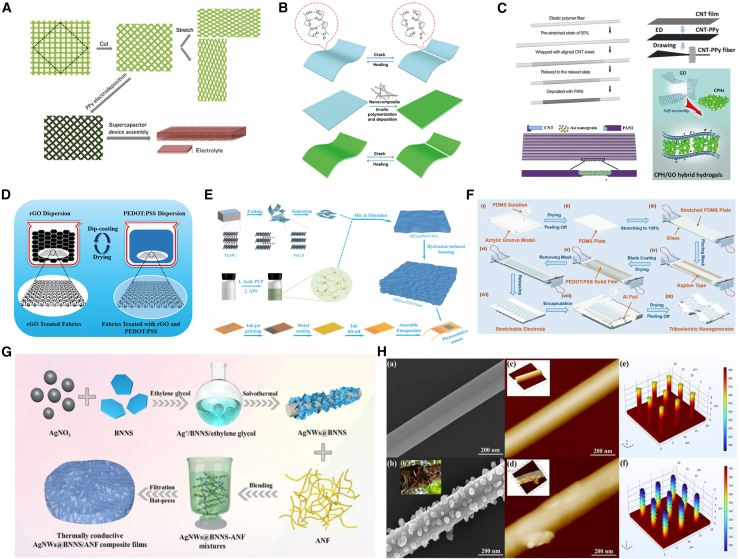
Application of conductive polymers and silver nanowires in smart wearable fibers and textiles (A and B) Schematic illustration of fabrication of stretchable steel mesh with PPy electrodeposition and schematic illustration of preparing the flexible healable all-in-one configured supercapacitor from the *in situ* polymerization. (C) Schematic illustration of fabrication of CNT/PANI fiber electrode, Schematic illustration of preparing the CNT-PPy composite fiber, Schematic illustration of fabrication of PANI@Au@CNT sheet, and Schematic illustration of CPH/GO hybrid hydrogels formation.^47^ Copyright (2019), with permission from Springer Nature. (D) Schematic of the fabrication strategy of CGPs.^49^ Copyright (2020), with permission from Royal Society of Chemistry. (E) Design and assembly of piezoresistive sensors based on MXene/PANI foam. The synthesis process of MXene (Ti_3_C_2_T_x_) colloidal solution. Synthesis procedures of PANI solution, Preparation process of MXene/PANI foam, and the fabrication steps of interdigital electrode and sensor assembly.^50^ Copyright (2022), with permission from John Wiley and Sons. (F) Schematic diagram of fabrication process flow of the stretchable and transparent wrinkled PEDOT:PSS film based triboelectric nanogenerator (WP-TENG).^51^ Copyright (2018), with permission from John Wiley and Sons. (G) Schematic diagram for the fabrication of thermally conductive AgNWs@BNNS/ANF composite films. (H) (a-f) AgNWs@BNNS SEM images, AFM images of fillers, and overall temperature distribution through finite element analysis.^52^ Copyright (2022), with permission from John Wiley and Sons.

Conductive polymers, considered organic polymers capable of conducting electricity, exhibit conductive or semiconductive behavior.^31^ As shown in Figures 4A–4C, there are currently over 25 known conductive polymers. They form an interesting class of materials that combine some of the mechanical properties of plastics with those of metals.^46^^,^^47^^,^^48^ These polymers have become a popular choice for conductive materials because they are lightweight and cost-effective, exhibiting relatively high adjustable conductivity (thanks to doping processes), flexibility, biocompatibility, can be customized to have sensing and actuation functions, and are easy to prepare. The biggest advantage of conductive polymers is their solvability, mainly through the dispersion of auxiliary agents in the aqueous phase (Figure 4D).^49^ They can be applied to energy storage and conversion devices, fuel cells, supercapacitors, adsorbents, conductive inks, multiphase catalysts, metal corrosion prevention, anti-static packaging, electrostatic discharge (ESD) control, EMI shielding applications, intelligent membrane technology, etc.

Intrinsic conductive polymers (ICP), also known as conjugated polymers and synthetic metals, have excellent electrical and optical properties. Different types of ICPs can be prepared, with conductivity ranging from 10^−10^ to 10^5^ S·cm^−1^. The most attractive among these polymers is poly (3,4-ethylenedioxythiophene) (PEDOT), one of the derivatives of polyaniline (PANI), polypyrrole (PPy), and polythiophene (PTh). Although these intrinsically conductive polymers (e.g., polyaniline, polypyrrole, etc.) are easy to synthesize because of their high electrical conductivity and environmental stability, the materials themselves are brittle and insufficiently flexible, resulting in poor mechanical properties. At the same time, in order to improve the conductivity of the material, it is often necessary to add a large number of conductive polymers, but conductive polymers added too much or unevenly dispersed, will cause damage to the continuity and integrity of the polymer matrix, resulting in a decline in mechanical properties.

PANI has attracted widespread attention due to its thermal and chemical stability.^53^^,^^54^ Its production cost is low, and it can be easily doped with inorganic and organic acids to prepare conductive materials and further improve conductivity. As shown in Figure 4E, Yin et al.^50^ realized MXene/PANI foam with 3D porous structure by using the steam induced foaming method. Based on this structure, a flexible piezoresistive sensor was manufactured. It exhibits high sensitivity (690.91 kPa^−1^), fast response, and excellent fatigue resistance (10000 cycles). The pressure sensor based on MXene/PANI foam can quickly detect tiny pressure and can be further used for human activity and health monitoring.

PPy, as a conductive polymer, is widely used as an electrode material for supercapacitors due to its low cost, environmental friendliness, easy preparation, and good redox properties. However, the poor conductivity, low solubility in common solvents, and unstable structure of PPy limit its practical applications. Liang et al.^55^ successfully prepared for the first time an independent self-supporting, adhesive free flexible polypyrrole: poly (styrene sulfonate) sodium polystyrene sulfonate/cellulose nanopaper electrode (PPy:PSS/PNP) using a low-cost, simple, and fast vacuum filtration method. Multilayer structured cellulose nanopaper has a high surface area and good mechanical strength, which not only provides a high electroactive area and shortens the diffusion distance of electrolyte ions, but also prevents the volume expansion/contraction of PPy during charging/discharging processes. The optimized PPy: PSS/PNP exhibits a high specific capacitance of 3.8 F·cm^−3^ (corresponding to 475 F·cm^−3^ and 240 F·g^−1^) and good cycling stability (80.9% capacitance retention after 5000 cycles) at 10 mV·s^−1^.

Sodium polystyrene sulfonate (PSS), a water dispersible polyelectrolyte, solves the solubility problem of PEDOT: PSS is used as a charge balancing counter ion in the oxidative polymerization process of 3,4-ethylenedioxythiophene (PEDOT) monomer. This resulted in the polymer complex poly (3,4-ethylenedioxythiophene) - poly (styrene sulfonate) (PEDOT: PSS).^56^ Polyionic complexes can be easily dispersed in water as colloidal gel particles with diameters of tens of nanometers, and have high stability. As shown in Figure 4F, Wen et al.^51^ fabricated a transparent and stretchable PEDOT: PSS electrode friction nanogenerator (WP-TENG), which exhibited good stretchability and transparency, with a maximum tensile strain of ≈100%. In addition, by being attached to human skin, WP-TENG can be used as an active human motion monitoring sensor. It can detect the bending angle of the elbow and joint by analyzing the peak value of the voltage output, and monitor the motion frequency by counting the peak value. WP-TENG’s excellent energy harvesting and active sensing capabilities, as well as outstanding transparency and stretchability, demonstrate its development prospects in new applications such as human-computer interaction, electronic skin, soft robots, wearable electronics, etc.

### Silver nanowires

Metal nanomaterials are an important component of nanomaterials and metal materials, as they fully combine the high conductivity and thermal conductivity of metal materials with the unique properties of nanomaterials. They have a wide range of applications in flexible wearable fiber electronic devices, mainly including zero-dimensional materials and one-dimensional materials.^57^^,^^58^^,^^59^^,^^60^^,^^61^^,^^62^ Among them, zero-dimensional materials are mainly composed of metal nanoparticles, such as copper nanoparticles; 1D materials are mainly composed of metal nanowires, such as silver nanowires (AgNWs).^63^^,^^64^^,^^65^^,^^66^^,^^67^ One-dimensional metal nanomaterials with a diameter less than 100 nm and a large aspect ratio have become a hot research topic in the field of smart wearables in recent years.^68^^,^^69^^,^^70^

As shown in Figures 4G and 4H, Han team^52^ prepared a multifunctional thermal conductive composite film based on AgNWs using BNNS ANFs as the matrix and AgNWs as the reinforcing functional material. By using chemical decomposition and *in situ* growth methods, the composite film has excellent electrical and thermal conductivity, excellent fracture strength (136 MPa), and flexible, sensitive, and stable strain, electrothermal, and photothermal sensing properties, which can play an important role in the fields of communication materials and intelligent sensors. Ma team^71^ prepared a highly elastic conductive fiber by adding zero-dimensional silver nanoparticles to polyurethane fibers through chemical synthesis. The resistance of AP (silver polyurethane) fibers increases with the increase of tensile length and temperature, as well as the elastic coefficient and Young’s modulus at different temperatures. The stress-strain also varies. AgNWs add conductivity to polyurethane fibers, and the combination of the two increases the tensile strength of the fibers. Li et al.^72^ prepared AgNWs using a liquid-phase polyol method, and then encapsulated them on the surface of an elastic polyurethane film (modified by immersion coating with graphene) using magnetron sputtering to prepare thin-film electronic devices. Zero-dimensional silver nanoparticles (AgNPs) are small in size and easy to disperse. They are uniformly coated on the surface of polyurethane films, greatly reducing the resistance of electronic devices to only 10 Ω·sq^−1^, and due to their extremely high sensitivity, strain based human motion sensing can be achieved. They can be mass-produced due to their simple process and low cost, and their high stability can increase their service life.

AgNWs have excellent electrical conductivity, but they are expensive to synthesis, have poor durability, and are prone to oxidization in air, which decreases their electrical conductivity, so they often need to be encapsulated, but it will have an impact on the breathability of smart textiles, which hinders their application in the field of smart textiles.

## Smart wearable fibers and textiles

### Conductive fibers

**Figure 5:**
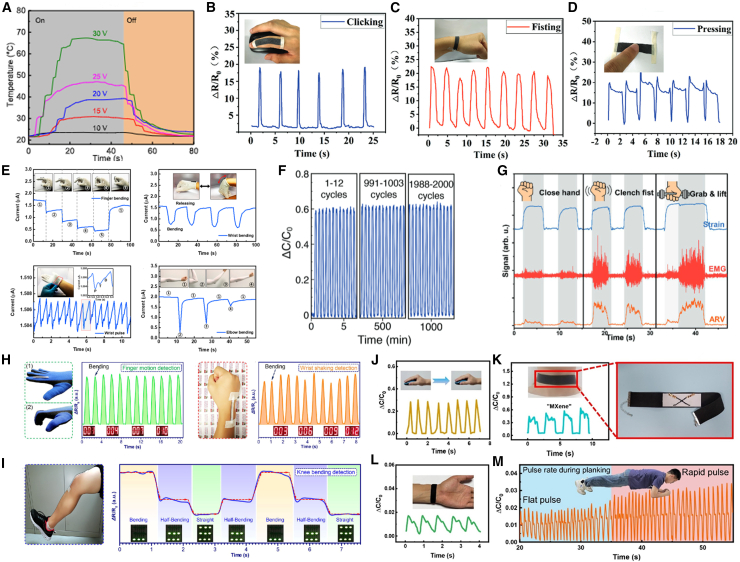
Smart wearable fibers and textiles for conductive fibers and personal healthcare sensors (A) The ANF/CNT/PPy aerogel fiber temperature−time curve.^73^ Copyright (2022), with permission from American Chemical Society. (B–D) Wearable applications in human motion detection of smart AMGP textile as strain sensors. Real-time relative change of resistance when detecting the movements of human body. Clicking, fisting, and pressing.^74^ Copyright (2023), with permission from John Wiley and Sons. (E) Detection of bio signals with the strain sensor driven by a supercapacitor. Current change with finger bending, wrist bending, wrist pulse, and elbow bending.^75^ Copyright (2019), with permission from American Chemical Society. (F) Cyclic stability of the sensor based on relative capacitance change at 14.1% strain for 2000 cycles.^76^ Copyright (2019), with permission from John Wiley and Sons. (G) Collected signals corresponding to closing hand, clenching fist, and grabbing and lifting, showing the combination of strain and EMG signals enable the recognition of different activities.^21^ Copyright (2022), with permission from John Wiley and Sons. (H and I) Collected signals corresponding to closing hand, clenching fist, and grabbing and lifting, showing the combination of strain and EMG signals enable the recognition of different activities.^77^ Copyright (2019), with permission from American Chemical Society. (J–M) The PDMA-F as an epidermal sensor detecting the human movements that click the mouse. The capacitance signal change of the PDMA-F based pressure sensor at the neck when pronounced “MXene”. The enlarged image shows the structure of the PDMA-F based pressure sensor on the wearable collar, The PDMA-F sensor accurately detect human pulse pressure, The sensing performance of PDMA-F epidermal sensor during the volunteer’s plank exercise under different intensities.^78^ Copyright (2024), with permission from American Chemical Society.

Nanocomposite conductive fibers have braiding and high conductivity, which can meet the requirements of flexible wearable devices. Huang et al.^73^ prepared light porous ANFs and CNTs composite aerogel fibers by wet spinning, and coated the surface with polypyrrole (PPy). ANF/CNT/PPy aerogel fiber has low density (56.3 mg·cm^−3^), conductivity (6.43 S·m^−1^), tensile strength (2.88 MPa) and long cycle life (1000 cycles). ANF/CNT/PPy aerogel fiber shows resistance response to pressure and can be used for pressure sensor and information transmission. In conclusion, the prepared ANF/CNT/PPy aerogel fiber has broad application prospects in human health detection, sports monitoring and other fields (Figure 5A).

Li et al.^79^ prepared aramid nanofibers/modified carbon nanotubes ultralight, flexible and strong aerogel fibers by surface wet spinning and freeze-drying technology. Aerogel textiles have excellent thermal insulation performance in the temperature range of −30∼200°C, low thermal conductivity of 42–60 mW·mK^−1^, and efficient electromagnetic shielding performance. The shielding value exceeds 30 dB. Aerogel textiles show great application potential as heat insulation and electromagnetic shielding equipment in harsh environments. In addition, Liu et al.^80^ prepared core-shell fibers with MXene as the core and ANF as the shell using wet spinning, which have high conductivity (3.0 × 10 ^5^ S·m^−1^), super toughness (48.1 MJ·m^−3^), high strength (502.9 MPa), and environmental stability. In addition, weaving this core-shell structure into fabric has an electromagnetic shielding effectiveness of 83.4 dB at a certain thickness (213 μm), which has good application prospects in the fields of electromagnetic shielding and wearable electronic devices. Li et al.^81^ prepared graphene fibers with high mechanical flexibility and self-cleaning function through wet spinning technology, and by modifying the conductive fibers with phase change materials, an intelligent temperature regulating fiber based on graphene was developed which can store and release heat through solid-liquid or liquid-solid phase transition when the temperature rises or falls, maintaining a suitable temperature for the human body. Afroj et al.^82^ successfully solved the problem of graphene fabric not being resistant to water washing by using a coated roller press method to prepare a graphene conductive sensor. The resistance value of the graphene sensor is as low as 11.9 Ω·sq^−1^, which has extremely high conductivity. After being packaged and protected, it can still maintain good conductivity even after 10 washing machine washes. The Jamali team applied a precursor solution of polyethylene oxide (PEO) – methyl lead bromide (MAPbBr_3_) onto CNTs-based fibers through a layer-by-layer solution coating method, successfully preparing carbon nanotube based luminescent fibers.^32^ The surface layer of the luminescent fiber is formed by coating silver nanowire dispersion after cooling and annealing, and the core is composed of highly elastic flexible fibers and CNTs fibers. Organic inorganic composite layers are alternately added between the core and the surface layer, adding many functions to the luminescent fiber.

Conductive fibers exhibit excellent electrical conductivity, flexibility and mechanical properties by compositing nanomaterials such as graphene, carbon nanotubes, MXene, conductive polymers and silver nanowires with the fiber matrix. These fibers are prepared using a variety of preparation methods, such as electrostatic spinning, wet spinning, coating, and co-blending spinning, which enable uniform dispersion of nanomaterials and optimization of fiber properties (Table 1). Graphene and carbon nanotubes are commonly used to make high-strength, highly conductive composite fibers due to their high conductivity and mechanical strength; MXene is suitable for sensors and electromagnetic shielding due to its 2D structure and high specific surface area; Conductive polymers (e.g., polyaniline and polypyrrole) are low-cost and flexible, which makes them suitable for large-area applications; and silver nanowires are used to make transparent conductive by virtue of their high conductivity and transparency fibers (Table 2).

**Table 1:** Comparison of advantages and disadvantages of different nanomaterials

Nanomaterials	Advantage	Disadvantage
Graphene	• Highly conductive • High mechanical strength • High thermal conductivity • Good chemical stability	• High cost of making single-layer graphene • Graphene flakes are easily stacked and poorly dispersed • Fiber integration is difficult
CNTs	• Highly conductive • High mechanical strength • High thermal conductivity • Good chemical stability • Easy fiber consolidation	• High cost of preparation of single-walled carbon nanotubes • Easy to agglomerate and poorly dispersed
MXene	• Good electrical conductivity • Low cost • Easy fiber consolidation	• General mechanical properties • General environmental stability • Less conductive than graphene and carbon nanotubes
Conductive polymers	• Low cost • Easy fiber consolidation	• Poor conductivity • Poor mechanical properties • Poor environmental stability (easily oxidized)
AgNWs	• Highly conductive • Good flexibility • Easy fiber consolidation	• High cost • Poor chemical stability • Weak mechanical properties

**Table 2:** The synthesis and application of conductive nanomaterials

Conductive nanomaterials	Synthesis method	Applications in smart wearable fibers and textiles
Graphene	• Mechanical stripping • Liquid phase stripping • Chemical vapor deposition • Epitaxial growth • Redox methods	• Flexible sensors • Lightweight supercapacitors • Thermoregulatory fabrics • Electrothermal fibers/fabrics
CNTs	• Chemical vapor deposition (CVD) • Laser ablation • Arc discharge	• High-sensitivity strain sensors • Woven into smart fabric circuits • Military/medical protective suits • Thermal management
MXene	• Chemical etching • Chemical vapor deposition (CVD) • Electrochemical stripping	• EMI shielding textiles • Flexible supercapacitors • Pressure sensors • Thermal management
Conductive Polymers	• Chemical polymerization • Electrochemical polymerization	• Stretchable electrodes • Transparent heating films • Flexible Sensors • Thermal management • Friction nanogenerators
AgNWs	• Chemical reduction • Polyol method • Hydrothermal method • Photoreduction technology	• Transparent conductive fabrics • Flexible sensors • Electromagnetic shielding • Electrothermal fibers/fabrics • Thermal management • Friction nanogenerators

Conductive fibers are widely used in smart textiles, flexible electronic devices, electromagnetic shielding and energy storage. For example, graphene fibers can be used for temperature regulation and health monitoring in smart clothing, carbon nanotube fibers can be used for electromagnetic shielding clothing, MXene fibers can be used for biosensors, conductive polymer fibers can be used for large-area flexible displays, and silver nanowire fibers can be used for transparent conductive films and wearable devices. These fibers not only enhance the functionality of traditional textiles, but also provide new solutions for lightweight and flexible electronic devices.

### Personal healthcare sensors

The whole-body motion signals of the human body can provide rich health information and diverse data for comprehensive monitoring of human condition and physical rehabilitation. Traditional sensors can only detect small strain behaviors and cannot accurately identify complex human motion information. Therefore, flexible strain sensors can be installed in different parts of the human body, and monitoring and recognition of human motion signals can be achieved through the different electrical signal waveforms fed back by the sensors to the strain behavior.^83^^,^^84^^,^^85^ For human motion monitoring, sensors not only need to have high sensitivity, but also a large strain testing range. Detecting bending movements of the elbow and knee joints requires strain sensors to operate normally over a large monitoring range. Meanwhile, flexible strain sensors can also detect and recognize subtle movements such as swallowing and speaking. The motion monitoring function of flexible strain sensors will play an important role in the future human application field of intelligent wearable electronic devices.^86^

Chen et al.^74^ used MXene as an inducer to interconnect silver nanowire (AgNW) cores with graphene sheaths, forming a stimulus responsive core sheath dual network structure. The fibers exhibited reliable responses to various mechanical/electrical/optical stimuli even under large mechanical deformations (100%). This core sheath type conductive fiber smart textile can seamlessly adapt to human movement and convert these mechanical deformations into precise medical monitoring characteristic signals with fast response (440 ms), which has great prospects in personalized healthcare and thermal management as shown in Figures 5B–5D. Park et al.^75^ integrated supercapacitors and strain sensors into a liquid metal interconnect integrated textile system. The system can successfully detect joint motion and strain caused by wrist pulses. It demonstrates the high feasibility of using the prepared scalable integrated textile system for real-time health monitoring of daily wearable devices (Figure 5E). Uzun et al.^76^ coated modified cellulose yarn with MXene, and after washing 45 times at 30°C–80°C, the MXene loading remained almost unchanged. And as a pressure sensor, volunteers wear it on their wrists to clearly recognize the pressure of touch. This is due to high sensor sensitivity (measurement coefficient 6.02), wide sensing range (up to 20 compressions), and good cycling stability (2000 cycles at 14% compression strain). It provides a feasible approach for the multifunctional integrated application of MXene in flexible electronic devices (Figure 5F). As shown in Figure 5G, the Liang team^21^ prepared hydrophilic graphene conductive materials using natural sericin assisted methods to overcome the defects of graphene’s lack of hydrophilicity, difficulty in dispersion, and tendency to aggregate. Due to its hydrophilic nature, the flexible electronic fabric prepared using this hydrophilic graphene conductive material has high breathability and water resistance, and can be used as a sensor to convert complex mechanical signals of human activities into electrical signals, successfully achieving human motion detection and health monitoring. It is an important research direction in the field of intelligent wearables. The Qi team^33^ utilized the high elasticity and conductivity of nickel-plated cotton fibers as the core layer, and prepared CNTs-based nanofiber yarns through electrospinning. This yarn has a core-shell structure, with a shell layer of polyurethane (PU) nanofibers embedded with CNTs. Therefore, the sensing sensitivity of this yarn is extremely high, and it has extremely high comfort. It can closely adhere to human skin without any foreign object sensation, making it an ideal material for flexible electronic fabrics. Appiagyei et al.^87^ used magnetron sputtering to deposit nickel and nickel oxide on the surface of fabrics to prepare a temperature sensor. The linearity (*R*^2^) of the temperature sensor can reach 0.9852. Due to the Ni/NiO double-layer thin film electrode formed by nickel and nickel oxide, the sensor has strong mechanical stability and can maintain high conductivity even after 16000 mechanical bending deformations. MXene/PU composite fibers exhibit sensing properties of GF∼12900 and∼152% high strain. When the MXene load is less than∼9.1wt %, the stress-strain curve exhibits three different behaviors, namely initial stiffness response, strain induced softening, and strain induced hardening. Using a knitting machine to weave MXene/PU fibers can produce sensors that surround human joints, such as wrist guards, elbow guards, etc., transmitting motion signals from various joints of the human body. As shown in Figures 5H–5I, the Liao team^77^ prepared flexible strain sensors on leather using LDW technology. The maximum strain coefficients for tension and compression of the sensor are 2009.5 and 50.9, respectively, and the minimum detectable strain is 0.005%. It can maintain mechanical stability throughout 10000 bending cycles. Hou et al.^78^ used interface induced welding of silver nanowires (AgNWs) and adhesive polydopamine functionalized MXene (PDM) as interface solder to assemble AgNWs along fibers. They developed a super stretchable conductor with a core sheath heterojunction interlocking structure, which has both high mechanical stretchability and high conductivity (1.13 × 10^5^ S·m^−1^), and can maintain stability even under large mechanical deformation (300%) (ΔR/R_0_ < 0.19). Super stretchable conductive fibers have broad prospects in the field of soft electronics, as shown in Figures 5J–5M.

Different sensing materials have different advantages for smart sensing. MXene-based flexible sensors have fast response speed but are easily oxidized with instability. AgNWs achieve suitable responsiveness and high conductivity, but the cost is high, and it is easily oxidized, and the environmental stability is poor. CNTs enable detection over a wide strain range, but their utility is limited by slow response time and environmental adsorption effects.

### Joule heating thermal management

**Figure 6:**
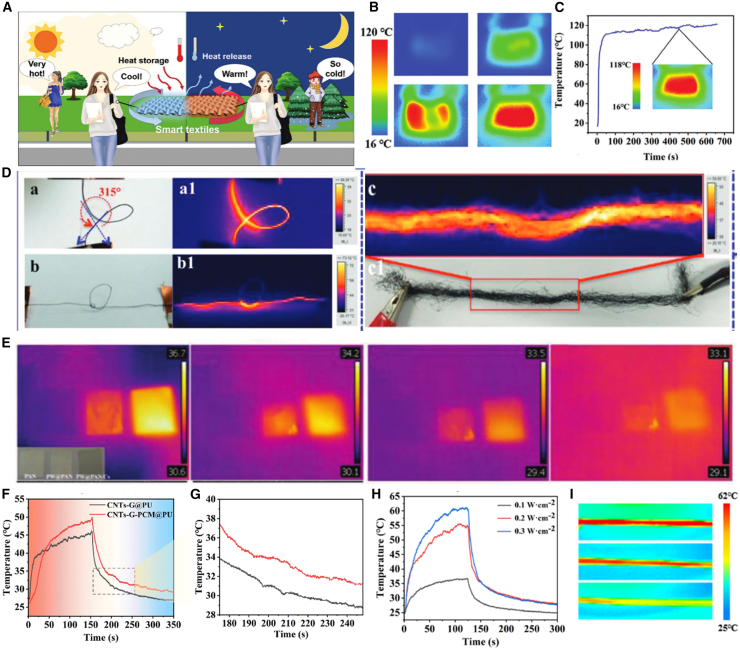
Smart wearable fibers and textiles for personal thermal management (A) Schematic diagram of photothermal experiment. (B and C) The corresponding infrared thermograms at different voltages (B), and electro-response stability of smart AMGP textile under 4 V for 700 s (C), the inset is the IR image of the textile tested for 450 s^74^ Copyright (2023), with permission from John Wiley and Sons. (D) Photograph and IR images of the ASF by applying an input voltage about 30.^81^ Copyright (2018), with permission from John Wiley and Sons. (E) Thermographic images and temperature variations of the PAN, PW@PAN and PW@PAN/Cs textiles with durable radiation time of 1, 5, 15, and 30 s. Reproduced with permission.^90^ Copyright (2021), with permission from American Chemical Society. (F and G) The light stimuli response curve and the corresponding enlarged image of CNTs-G@PU and CNTs-G-PCM@PU. (H and I) The light stimuli response curves of smart responsive fiber and the corresponding infrared images.^91^ Copyright (2023), with permission from Elsevier.

The selection of conductive materials should comprehensively consider the compatibility between conductive components and substrate elastic fibers, so that flexible electronic fabrics have excellent carrier transfer channels, extremely high conductivity, ideal mechanical properties, and stretchability.^39^^,^^88^ In recent years, with the continuous emergence of nanotechnology and nanomaterial technology, nanomaterials have gained more and more advantages that can be supplied and adapted.^89^ Chen et al.^74^ designed The AMGP smart fabric has excellent electrical and thermal response performance, electrical stimuli are applied to the smart AMGP textile, the corresponding temperature will raise up as a response due to the excellent Joule heating effect of the core–sheath heterogeneous interlocked conductive network. The temperatures of smart AMGP textile response to electrical stimuli are 25.5°C (1 V), 42.3°C (2 V),75°C (3 V), and 113°C (4 V) respectively, While increased rapidly along with the stepwise voltage and maintaining the stable temperature under the corresponding stimuli, as shown in Figure 6A–6C. Due to the high conductivity and elasticity requirements of flexible wearable electronic fabrics, nanomaterials are often chosen to construct flexible conductive networks on fiber/fabric surfaces, such as carbon-based nanomaterials, 2D layered materials nitrides, and metal nanomaterials,^81^ as shown in Figure 6D. Efficient heating is achieved through the Joule heating effect generated by conductive nanomaterials (such as graphene, silver nanowires, etc.) when powered on. This type of material, with its nanoscale ultrafine structure and highly conductive network, can quickly heat up at voltages far below human safety.

The Levitt team^92^ mixed MXene with nylon (PA)/spandex (PU) and prepared nano conductive yarns using electrospinning. MXene/PU yarn has the characteristics of high elasticity and high conductivity, while MXene/PA yarn has the characteristics of high toughness and high tensile strength. It has broad prospects in the fields of heat storage temperature regulation, motion sensing, and personal health management. Ding’s team^36^ blended anisotropic thermal storage and temperature regulating materials with MXene and prepared composite nanofibers with unidirectional thermal conductivity through electrospinning technology. This fiber has anisotropy and can conduct heat unidirectionally, which is more conducive to keeping warm. Moreover, this fiber has broad prospects in the field of medical dressings, as its unidirectional thermal conductivity allows for the use of photothermal effects to kill recurrent cancer cells on the outside of the wound after surgery. This study provides new ideas for postoperative recovery and medical rehabilitation, and has broad application prospects in the field of biomedicine. The Yang team utilized the property of MXene having a large number of active groups to combine the active groups of MXene with those of oxidized graphene to form chemical bonds, and prepared MXene GO nanofibers by wet spinning method.^27^ Graphene oxide nanosheets are uniformly dispersed and arranged in an orderly manner between MXene nanosheets, with MXene as the matrix and a mass ratio of up to 95:5 to graphene oxide. This MXene based hybrid fiber has extremely high conductivity (2.9 × 10^4^ S·m^−1^), which can be further used to prepare strain sensors, supercapacitors, etc. The capacitance value can reach 586.4 F·cm^−3^, which is superior to pure reduced graphene fibers in terms of performance.

### Photothermal heating thermal management

Nanomaterials have opened up a new dimension of photothermal regulation for smart textiles, and their core principle relies on the material’s ability to capture and convert light energy. Through localized surface plasmon resonance (LSPR) or band transition effects, specific wavelength light energy (especially near-infrared light) can be efficiently converted into thermal energy, with a conversion efficiency of over 80%.

Thermal comfort is a fundamental requirement for all wearable related applications. For example, heated fabrics can be used in medical care because thermal therapy helps to open blood vessels, increase blood flow, and provide nutrients for wound healing. Due to the light-dependent properties of photothermal fabrics, they can be used for medical infrared lamp thermal therapy. Wearable electronic products based on intelligent temperature regulation can accurately identify and monitor human activities and health, and provide corresponding signal feedback in real time (Figure 6E). With the emergence and development of smart wearable electronic devices and healthcare systems, the strong demand for energy-saving and effective personal temperature regulation has attracted widespread attention to prevent thermal runaway and achieve thermal comfort for the human body during long-term wear.^90^^,^^93^^,^^94^^,^^95^ When people experience sudden cooling or drastic weather changes outdoors, long-term work in high temperatures or outdoor environments, and an imbalance in human health, thermal runaway can occur. On the other hand, during the operation of wearable electronic devices, heat will continue to be generated. If heat cannot be effectively dissipated, it will accumulate, leading to thermal runaway around the human body. Meanwhile, flexible batteries are often used in self-powered wearable electronic products, and sometimes encounter short circuit problems, leading to thermal runaway accidents. Due to the tight adhesion of installable, flexible, and wearable electronic products to human skin, uncontrollable temperature fluctuations during long-term wear may cause physical/psychological discomfort and even endanger life, such as high temperatures above 37.3°C–38.5°C or low temperatures below 35.0°C. Therefore, developing efficient human temperature control strategies and solutions to achieve thermal comfort is crucial in emerging wearable applications. He et al.^91^ successfully developed an elastic intelligent multi responsive PCM based fiber for efficient personal healthcare and thermal management through continuous wet spinning. The author embedded PCM microcapsules with dual responsive networks in elastic PU, spun them into smart fibers, and introduced high concentrations of carbon nanotubes/graphene onto the PU surface through PEDOT: PSS bonding, forming a stimulus responsive level network to prepare smart fibers. Smart CNTs-G-PCM@PU fibers not only have good response capabilities to various stimuli such as electricity, heat, temperature, and light, but can also convert them into potential thermal energy during the response process, and maintain good stimulus response even when the mechanical elongation exceeds 200%. They can be further developed for self-sufficient personalized medical and thermal management, and have great prospects for the next generation of wearable electronic products and smart textiles, as shown in Figures 6F–6I.

As the above two important heating technologies, Joule heating and photothermal heating have their own unique advantages, disadvantages, and application scenarios. Joule heating generates heat through electric current, which has the advantages of rapid warming, high efficiency, and precise control, but requires conductive materials and high energy consumption, and is suitable for electric heating fibers in smart wearable textiles, such as rapid warming of warm clothing. Photothermal heating, on the other hand, utilizes light energy converted into heat energy, which is a non-contact and precisely positioned heating method, suitable for solar-powered or micro-nano-scale heating, but with lower energy conversion efficiency and dependent on light conditions. In the field of smart wearable, photothermal heating can be used for solar-powered self-heating clothing or localized thermal therapy devices. Both show potential for application in smart textiles, with Joule heating suitable for fast and uniform heating scenarios, while photothermal heating is more suitable for solar energy utilization or localized heating needs (Table 3).

**Table 3:** Comparison between joule heating and photothermal heating

	Joule heating	Photothermal Heating
Heating efficiency	High	Low
Power requirements	High power	Low power
Response time	Fast response time	Slower response time
Application scenario	Wearable scenarios requiring fast, precise temperature control Advantage: fast, precise temperature control Disadvantage: High power demand, Prolonged use may result in high energy consumption	Outdoor wearable scenarios with solar radiation Advantage: Environmentally friendly and low energy consumption Disadvantage: Efficiency and response time limited by light conditions
Key factor	Improved electrical conductivity	Improve light absorption and increase the efficiency of light and heat conversion

### Electromagnetic interference (EMI) shielding

With the proliferation of electronic devices, EMI shielding in wearable textiles is critical to protect humans from harmful radiation and ensure device reliability. Nanomaterials with high conductivity and tunable structures are ideal for lightweight and flexible EMI shielding textiles.

**Figure 7:**
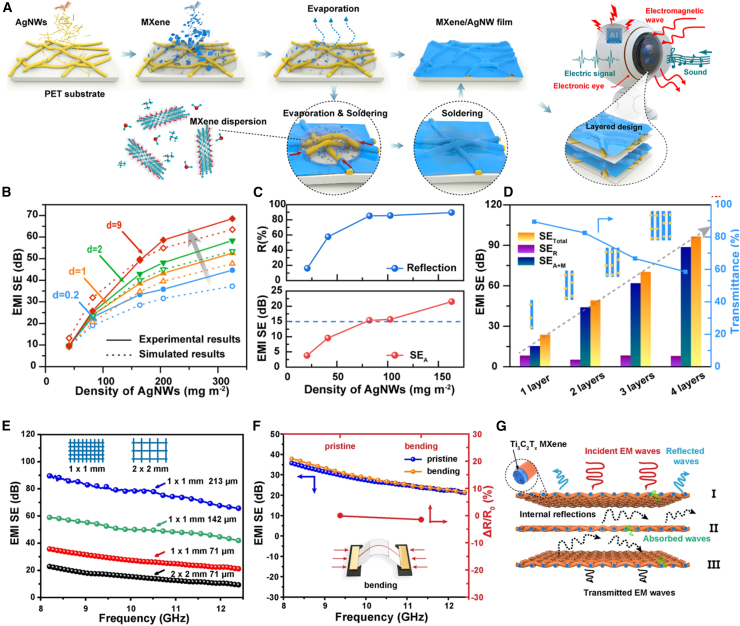
Smart wearable fibers and textiles for electromagnetic interference shielding (A) Schematic illustrating the fabrication procedure of a transparent MXene/AgNWs film. (B) Comparison of experimental and simulated EMI shielding performances of d-MA films with different total AgNW densities and gap distances. (C and D) Plots of (C) reflection and SEA of a constituent layer with different AgNW loadings. (D) EMI shielding performances of stacked structures with different MA10/82 layers (d = 9 mm).^42^ Copyright (2020), with permission from American Chemical Society. (E) Plots of EMI SE versus different mesh grids and thicknesses of the 17–1.1–50 M textile. (F) Changes in resistance and EMI shielding performance after 5,000 cycles of bending. (G) Possible EMI shielding mechanisms of the ANF@M textiles.^80^ Copyright (2022), with permission from Springer Nature.

Chen et al.^39^ prepared flexible electronic devices with good mechanical properties, environmental stability, and sensitive pressure response performance by MXene-welded AgNW films, and this wearable product showed high EMI shielding performance (34 dB) with a shielding efficiency of 49.2 dB, while its light transmittance was as high as 83%, which is expected to be used in next-generation electronic products to realize many potential applications, as shown in Figures 7A–7D. Wang et al.^58^ prepared flexible and mechanically performant composite films of silver nanowires and biopolymers, AgNWs@BNNS, with EMI shielding efficiencies (SE) of up to 65 dB in the X-band (8–12.4 GHz). Coaxial fibers developed by Liu et al.^80^ based on Aramid Nanofibers (ANF) and MXene not only have good flexibility and mechanical properties but also the shielding efficiency (SE) of the fibers can reach more than 60 dB in the X-band (8.2–12.4 GHz), which can block more than 99.999% of the electromagnetic waves, and has a good application prospect in the field of flexible electronic devices and wearable devices as well as aerospace, as shown in Figures 7E–7G.

## Challenges

Smart wearable fibers and textiles take advantage of the unique properties of nanomaterials (e.g., high sensitivity, flexibility, and electrical conductivity) and show great potential for health monitoring, human-computer interaction, and more. These properties enable smart wearable devices to conform the human body seamlessly, monitor physiological signals in real time, provide a comfortable wearing experience, and enable efficient energy management. However, certain challenges still exist for smart wearable fibers and textiles at present.

Although nanomaterials, with their small size and large specific surface area, can easily interact with human tissues and show good biocompatibility in the short term, their compatibility for long-term use still needs to be further verified. For example, materials such as CNTs and graphene may cause inflammatory reactions or tissue damage in long-term use. Meanwhile, some nanomaterials may be potentially toxic (e.g., metal nanoparticles), and the use of these nanomaterials may accumulate inside the human body and cause damage. The complex processing and synthesis methods of nanomaterials lead to high production costs, which severely limit their applications, such as the high cost of preparing graphene with single lamellar layers and CNTs with single walls. Meanwhile, the complex synthesis process of some nanomaterials requires strict control of the synthesis conditions to guarantee the homogeneity and consistency of the materials. For example, the etching process of MXene requires strict control of etching time and etching temperature. In addition, for conductive polymers, although their production cost is low, their mechanical properties are poor, so it is still a problem to safeguard the functional properties of nanomaterials while improving the wear resistance and durability of the materials in the production process. Also, the Joule heating and photothermal heating types of smart wearable devices are power and light dependent. In the case of joule-heated wearable devices, external power devices are required, which require a large amount of energy to increase the cost during use, while there are serious safety issues. Although the photothermal heating wearable device has low energy consumption, it has a serious dependence on light conditions, and at the same time, the conversion efficiency is lower than that of Joule heating. Therefore, it is necessary to prepare smart wearable devices with multi-response performance, but this will inevitably require the wearable devices to have more functions at the same time (e.g., stimulus-responsive performance of light, electricity, heat, and magnetism, etc.).

In addition, during long-term wearable applications, while considering the smart response performance of wearable devices, we should also pay attention to their abrasion resistance, stretchability, and water washing resistance, which is an inevitable requirement to ensure the long-term use of wearable devices. But at present, it is still a challenge to provide wearable devices with both the smart response performance and the safety and durability that traditional fabrics should be possessed. Therefore, when integrating nanomaterials into wearable devices, the comprehensive performance of both should be taken into account in order to prepare multifunctional, durable, and user-friendly wearable textiles. Finally, the current production methods make it difficult to realize the mass production of smart wearable products, such as coating and dip-coating technologies, although simple to operate and more suitable for large-area continuous production, suffer from the problems of uneven and poor stability of the coating, which may fall off when flexible electronic devices are repeatedly bent and rubbed. Printing technology, although can precisely control the pattern, suitable for the manufacture of textiles with specific functions, but the equipment requirements, high cost, and operational complexity. Although electrostatic spinning technology can prepare high-performance nanofibers, it has low production efficiency and is difficult to be used for mass production. And although wet spinning technology can achieve uniform dispersion of nanomaterials and is more suitable for large-scale production of continuous fibers, the production process requires a large number of solvents and the post-treatment process is complicated. Therefore, for industrial or practical production, it is also necessary to choose the appropriate production strategy according to the specific textile functional requirements to meet the requirements of mass production.

The development of environmentally friendly nanomaterials not only reduces the impact on the environment, but also improves the biocompatibility of the materials. Meanwhile, research on greener and more efficient synthesis process can reduce the harmful substances produced in the production process and prepare more efficient and durable nanomaterials. In addition, the preparation of smart wearable devices with multiple performance fusion can effectively improve the efficiency of energy utilization and reduce energy consumption and cost. Considering smart corresponding performance and durability comprehensively, more practical smart wearable textiles can be developed and fabricated. Therefore, in the future, multidisciplinary cross-collaboration of materials science, biomedicine, and AI is expected to solve the current problems and challenges faced by smart wearable fibers and textiles, so that wearable devices can provide people with healthier, more comfortable, and more environmentally friendly lifestyles.

## Conclusions

Nanomaterials have transformed traditional fibers and textiles into smart wearable systems with applications spanning personal healthcare, robotics, thermal management, energy harvesting and storage, and environmental monitoring. While challenges in durability and scalability persist, interdisciplinary innovations in material science and fabrication techniques promise to unlock next-generation smart wearable fibers and textiles. Continued research into biocompatible, self-healing, and energy-autonomous designs will drive this field toward widespread commercialization. While challenges remain, advancements in nanotechnology and material science continue to push the boundaries of what is possible. Future research should prioritize scalable, eco-friendly solutions to realize the full potential of these transformative materials. Collaborative efforts across nanotechnology, materials science, and textile engineering will be pivotal in realizing wearable systems that enhance healthcare, safety, and sustainability. Especially with the emergence of AI technology in recent years, the development of next-generation of intelligent wearable fibers and textiles using AI technology will further accelerate the rapid development of this field.

## Acknowledgments

This work was supported by the 10.13039/501100001809Natural Science Foundation of China (No. 52203061, 52303060), Young Talent of Lifting Engineering for Science and Technology in Shandong Province (Grant SDAST2024QTA066), 10.13039/501100007129Natural Science Foundation of Shandong Province (ZR2023QB046), the Opening Project of Textile Ecological Dyeing and Finishing Key Laboratory of Sichuan Province (Chengdu Textile College) (2024DF-A01), and the University Synergy Innovation Program of Anhui Province (No. GXXT-2023-096).

## Author contributions

The manuscript was written through contributions of all authors. All authors have given approval to the final version of the manuscript. L.Z., W.Z. and S.L. wrote the article. J.W. and Y.Y. checked the article and provided suggestions. J.M. proposed conceptual writing ideas and revised the article.

## Declaration of interests

The authors declare that we have no financial and personal relationships with other people or organizations that can inappropriately influence our work.
